# siRNA knockdown of mitochondrial thymidine kinase 2 (TK2) sensitizes human tumor cells to gemcitabine

**DOI:** 10.18632/oncotarget.4272

**Published:** 2015-06-15

**Authors:** Christine Di Cresce, Rene Figueredo, Mateusz Rytelewski, Saman Maleki Vareki, Colin Way, Peter J. Ferguson, Mark D. Vincent, James Koropatnick

**Affiliations:** ^1^ Department of Microbiology and Immunology, The University of Western Ontario, London, Ontario, Canada; ^2^ Department of Oncology, The University of Western Ontario, London, Ontario, Canada; ^3^ Department of Pathology, The University of Western Ontario, London, Ontario, Canada; ^4^ Department of Physiology and Pharmacology, The University of Western Ontario, London, Ontario, Canada; ^5^ Cancer Research Laboratory Program, Lawson Health Research Institute and London Regional Cancer Program, London, Ontario, Canada

**Keywords:** cancer, gemcitabine, thymidine kinase, deoxycytidine kinase, mitochondria

## Abstract

Nucleoside metabolism enzymes are determinants of chemotherapeutic drug activity. The nucleoside salvage enzyme deoxycytidine kinase (dCK) activates gemcitabine (2′, 2′-difluoro-2′-deoxycytidine) and is negatively regulated by deoxycytidine triphosphate (dCTP). Reduction of dCTP in tumor cells could, therefore, enhance gemcitabine activity. Mitochondrial thymidine kinase 2 (TK2) phosphorylates deoxycytidine to generate dCTP. We hypothesized that: (1) TK2 modulates human tumor cell sensitivity to gemcitabine, and (2) antisense knockdown of TK2 would decrease dCTP and increase dCK activity and gemcitabine activation. siRNA downregulation of TK2 sensitized MCF7 and HeLa cells (high and moderate TK2) but not A549 cells (low TK2) to gemcitabine. Combined treatment with TK2 siRNA and gemcitabine increased dCK. We also hypothesized that TK2 siRNA-induced drug sensitization results in mitochondrial damage that enhances gemcitabine effectiveness. TK2 siRNA and gemcitabine decreased mitochondrial redox status, DNA content, and activity. This is the first demonstration of a direct role for TK2 in gemcitabine resistance, or any independent role in cancer drug resistance, and further distinguishes TK2 function from that of other dTMP-producing enzymes [cytosolic TK1 and thymidylate synthase (TS)]. siRNA knockdown of TK1 and/or TS did not sensitize cancer cells to gemcitabine indicating that, among the 3 enzymes, only TK2 is a candidate therapeutic target for combination with gemcitabine.

## INTRODUCTION

Nucleoside-metabolizing enzymes are important determinants of the activity of antimetabolite chemotherapy drugs [1]. However, other than the use of inhibitors of catabolic enzymes [2], little has been done to manipulate metabolic pathways to improve drug efficacy. The nucleoside salvage enzyme deoxycytidine kinase (dCK) normally catalyzes phosphorylation of deoxycytidine to its monophosphorylated form [3]. In addition, dCK also activates gemcitabine (2′, 2′-difluoro-2′-deoxycytidine, dFdC; a deoxycytidine analog anti-metabolite anticancer drug) by catalyzing production of gemcitabine monophosphate and subsequent active metabolites [6]. However, dCK is negatively regulated by deoxycytidine triphosphate (dCTP) and increased dCTP diminishes the net cytotoxic, anticancer activity of gemcitabine.

Thymidine kinases 1 and 2 (TK1 and TK2) are salvage enzymes capable of phosphorylating cellular thymidine to generate deoxythymidine essential for DNA synthesis. Unlike cytosolic, cell cycle-dependent TK1, TK2 is cell cycle-independent and present in mitochondria. TK2, but not TK1, is promiscuous with respect to substrate [4–6]: it can, in addition to phosphorylating thymidine, phosphorylate deoxycytidine to generate dCTP that, in turn, can reduce gemcitabine activity. Thus, TK2 in human tumor cells could reduce gemcitabine effectiveness.

We previously reported that TK2 has potential as a therapeutic target in treatment of human cancers if its down-regulation by antisense is combined with widely-used cytotoxic anticancer agents and antisense molecules targeting thymidylate synthase (TS) [7]. In that scenario, antisense-reduced TS and TK2 combined to sensitize human tumor cells to the anti-TS drug 5-FUdR. Functions of TK2, including those distinct from cytosolic TK1, are not fully characterized. Inhibition of TK2 has potential to improve cancer therapy.

Gemcitabine is used to treat a variety of tumor types (lung, breast, ovarian, bladder and pancreas) [8]. Incorporation of gemcitabine triphosphate (dFdCTP) into DNA in place of dCTP during synthesis results in cell death due to termination of DNA synthesis [9] and gemcitabine diphosphate (dFdCDP) is thought to irreversibly bind the active M1 subunit of ribonucleotide reductase (RR) with therapeutic benefit [10]. dCK, in addition to phosphorylating deoxycytidine to generate dCMP, also mediates the initial activating phosphorylation of the anticancer drug gemcitabine (a prodrug) to produce gemcitabine monophosphate and subsequently active metabolites [11]. Under normal conditions of feedback regulation, deoxycytidine triphosphate (dCTP) negatively regulates dCK activity [12, 13]. Thus, the presence of a pool of dCTP represses dCK activity and gemcitabine activation.

The substrate promiscuity of TK2 suggests that TK2-mediated production of dCMP and the resulting increase in dCTP could contribute to dCK feedback inhibition that decreases gemcitabine activation and effectiveness. We hypothesized that: (1) TK2 contributes to cellular resistance to gemcitabine, and (2) antisense siRNA downregulation of TK2 will sensitize human tumor cells to gemcitabine. It is important to note that gemcitabine is a poor substrate for TK2 [14].

TK2 is a constitutively-expressed mitochondrial enzyme that participates in maintenance and production of mitochondrial DNA (mtDNA). Gemcitabine decreases mtDNA synthesis by inhibiting mitochondrial DNA polymerase holoenzyme (DNA polymerase gamma) [15]. Consequently, the potential for antisense-mediated reduction of TK2 to modulate mitochondrial function, increase mitochondrial damage, and improve gemcitabine cytotoxicity was explored.

We show for the first time that siRNA-mediated knockdown of TK2 sensitized TK2-expressing cancer cell lines (MCF7 and HeLa) to the anti-proliferative effects of gemcitabine *in vitro*. The effect was specific to TK2, as knockdown of other dTMP-producing enzymes (thymidylate synthase and TK1), alone or in combination with TK2 siRNA treatment, did not further sensitize tumor cells to gemcitabine beyond that induced by TK2 knockdown alone. Combined TK2 siRNA and gemcitabine treatment inhibited mitochondrial function.

## RESULTS

### TK2 is expressed in human tumor cells and decreased by siRNAs

**Figure 1:**
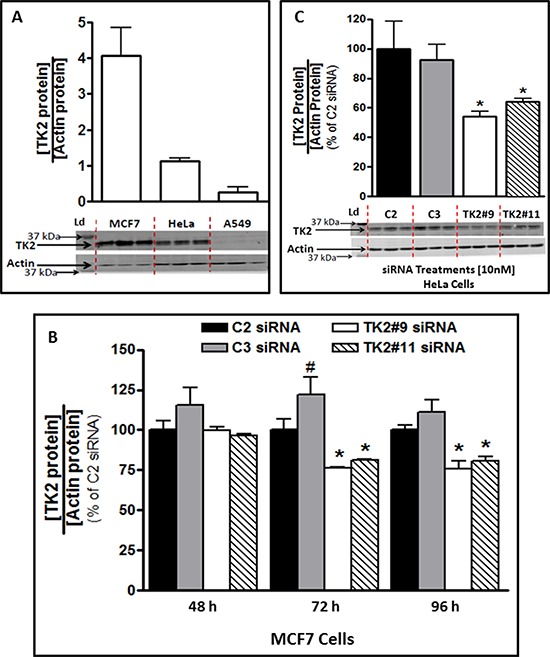
TK2 protein in MCF7 and HeLa cells is reduced by TK2 siRNA **A.** Basal TK2 in MCF7(TK2^HIGH^), HeLa(TK2^MEDIUM^) and A549(TK2^LOW^) cells (*n* = 9). **B.** Relative TK2 protein levels in MCF7 cells, 48–96 h post-transfection with 10 nM TK2 siRNA (see Supplementary Figure S1 for mRNA levels), and **C.** in HeLa cells, 96 h post-transfection with 10 nM TK2 siRNA. In both cases, relative TK2 protein is shown as a percent of the amount in cells transfected with control, non-targeting C2 siRNA. Bars represent means ± SEM (*n* = 9). In panels A and C, immunoblots show triplicate independent samples. *different from cells treated with C2 or C3 siRNA (*p* < 0.05, Student's *t* test or ANOVA). ^#^different from cells treated with C2 siRNA (*p* < 0.05, Student's *t* test or ANOVA).

MCF7, HeLa and A549 human tumor cell lines differed with respect to basal TK2 protein (Figure 1A) and are labeled TK2^HIGH^ (MCF7), TK2^MEDIUM^ (HeLa) and TK2^LOW^ (A549). A549 cells had only 7%, while HeLa cells had 30%, of the amount of TK2 in MCF7 cells.

TK2 siRNAs downregulated TK2 mRNA in MCF7 cells by 60–75% compared to control at 24–96 h post-transfection (Supplementary Figure S1). This translated to a decrease in TK2 protein of approximately 25% by 72 h and 96 h post-transfection (Figure 1B). HeLa cell TK2 protein was similarly reduced by siRNA treatment at 96 h post-transfection (Figure 1C). MCF7, HeLa, and A549 cells had similar siRNA transfection efficiencies (Supplementary Figure S2). Consequently, differences among cell lines caused by TK2 siRNA transfection are not attributable to differential transfection efficiency.

### Antisense knockdown of TK2 sensitized TK2^MEDIUM^ (HeLa) and TK2^HIGH^ (MCF7) cells, but not TK2^LOW^ (A549) cells, to gemcitabine

TK2-targeting siRNAs #9 and #11 target different portions of the TK2 mRNA sequence. TK2#9 siRNA and TK2#11 siRNA (compared to C2 and C3 non-targeting siRNAs, respectively), reduced TK2 in all 3 cell lines. The effect of antisense-mediated TK2 reduction on sensitivity to gemcitabine was assessed. To demonstrate siRNA-induced sensitization to gemcitabine, proliferation in the presence of combinations with gemcitabine were normalized to that of treatment with siRNAs alone (without gemcitabine). There was no significant inhibition of proliferation induced by control siRNAs alone (C2 or C3) or TK2-targeting siRNAs alone (TK2#9 or TK2#11) (Supplementary Figure 3).

**Figure 2:**
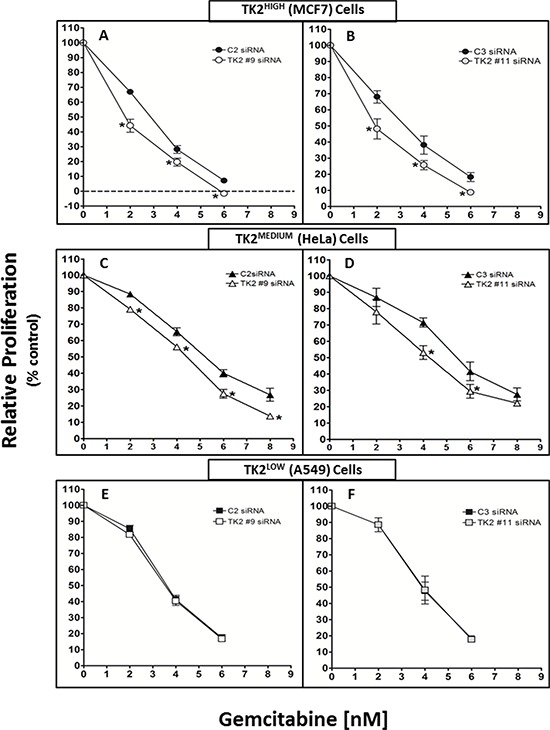
siRNAs targeting TK2 sensitize TK2-expressing human tumor cell lines to gemcitabine MCF7 **A, B.** HeLa **C, D.** and A549 **E, F.** cells were transfected with 10 nM C2, TK2#9, C3, or TK2#11 siRNAs and treated with gemcitabine as described in *Materials and Methods*. Proliferation was measured by cell counting at 96 h post-transfection. Data are expressed as a percent of the cell number after treatment with siRNA alone (without drug). Bars represent means ± SEM (*n* = 9). *different from cells transfected with control, non-targeting siRNA (*p* < 0.05, Student's *t* test).

TK2 siRNA sensitized TK2^HIGH^ MCF7 cells and TK2^MEDIUM^ HeLa cells to gemcitabine (Figure 2). TK2 knockdown in TK2^HIGH^ MCF7 cells by either of the two TK2 siRNAs enhanced gemcitabine-mediated reduction in proliferation by 30–50% (Figure 2A, 2B) and TK2^MEDIUM^ HeLa cells by 15–50% (Figure 2C, 2D), depending on the gemcitabine concentration. TK2 siRNA treatment did not sensitize TK2^LOW^ (A549) cells to any tested gemcitabine concentration (Figure 2E, 2F).

### Combined treatment with TK2 siRNA and gemcitabine decreased TK2 and increased dCK

**Figure 3:**
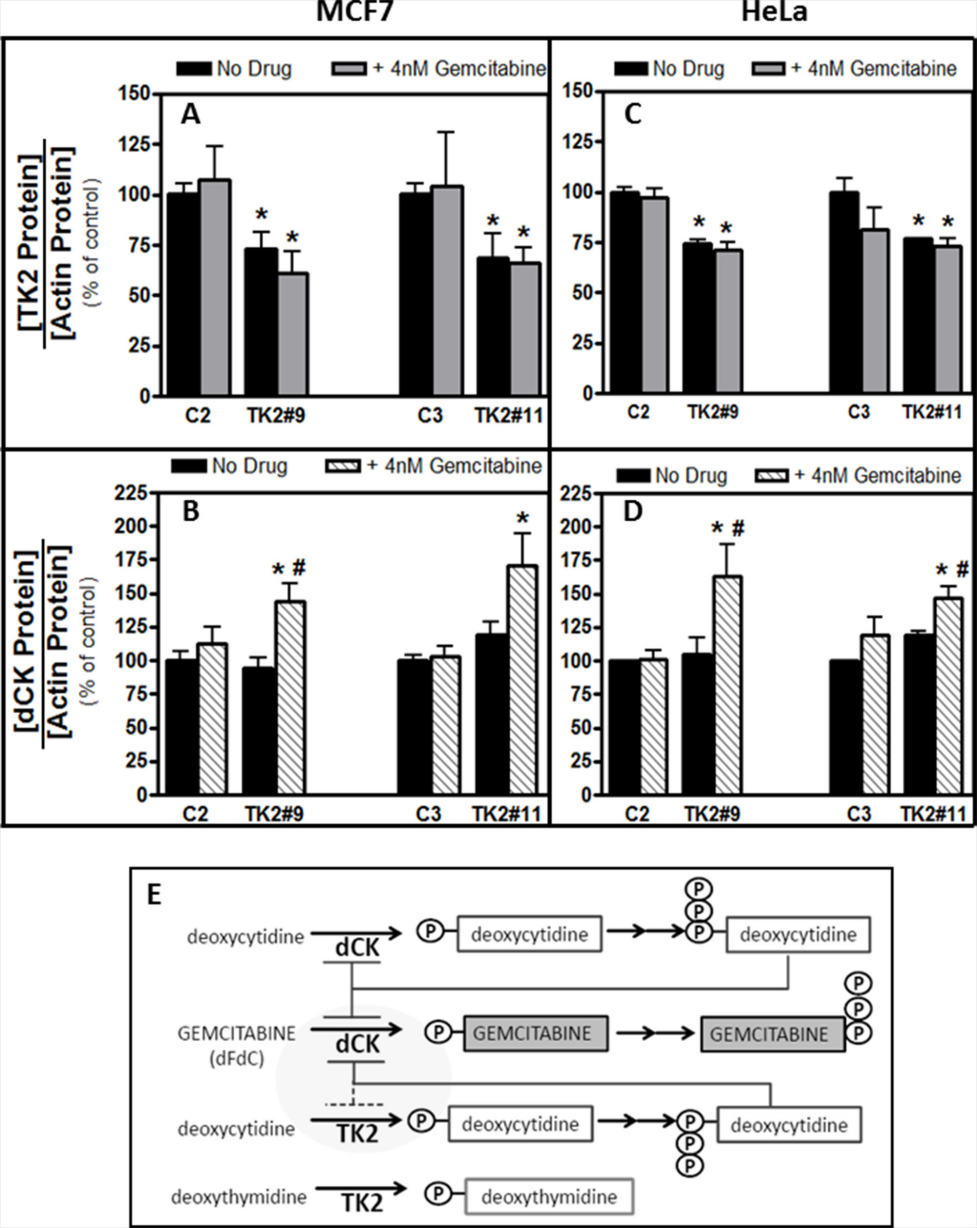
Combination of TK2 knockdown and gemcitabine treatment increase dCK in MCF7 and HeLa cells MCF7 **A, B.** and HeLa **C, D.** cells were transfected with C2, C3, TK2#9, or TK2#11siRNAs, treated with gemcitabine, and relative TK2 and dCK protein levels were measured 96 h post-transfection as described in *Materials and Methods* (see Supplementary Figure S4 for siRNA-mediated knockdown of TK2 mRNA in the same cells for which these data are shown). Bars represent means ± S.E.M for *n* = 8–9 samples. *different from cells transfected with control, non-targeting siRNA (*p* < 0.05, Student's *t* test). ^#^different from cells treated identically but without gemcitabine siRNA (*p* < 0.05, Student's *t* test). **E.** Proposed relationship between TK2, dCK and gemcitabine.

TK2^HIGH^(MCF7) and TK2^MEDIUM^(HeLa) cells were collected 96 h after transfection with siRNA and treatment with gemcitabine (4 nM) (Supplementary Figure S4). In both cell lines, with or without gemcitabine, TK2 mRNA and protein levels were decreased by TK2 siRNA (Supplementary Figure S4 and Figure 3A, 3C). In both of these TK2-expressing cell lines, combined treatment with TK2 siRNA and gemcitabine reduced TK2 protein levels and increased dCK protein levels (Figure 3A–3E).

### Sensitization effects were specific to TK2: siRNAs targeting other dTMP-producing enzymes did not sensitize to gemcitabine

**Figure 4:**
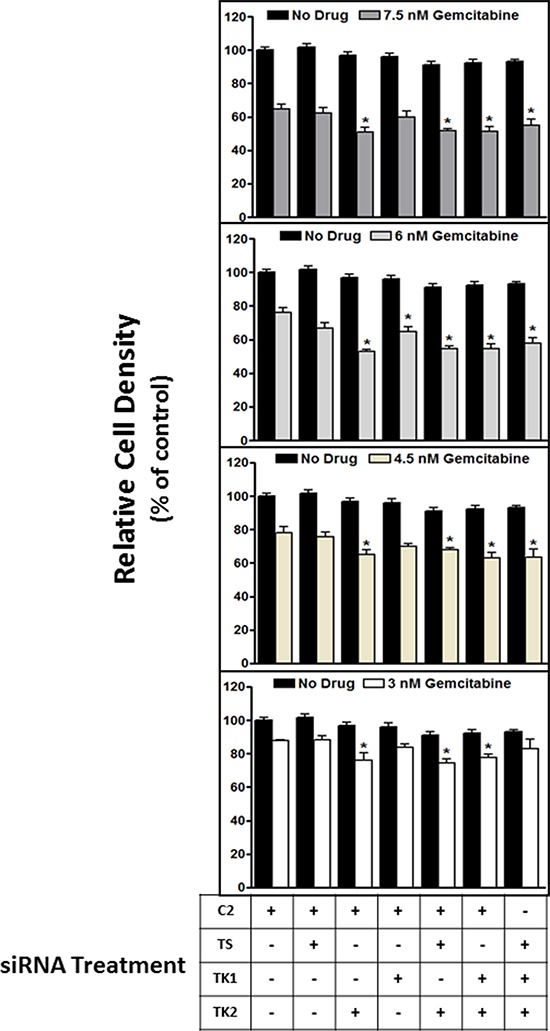
siRNA targeting of TK2, but not TS or TK1, contributes to sensitization to gemcitabine HeLa cells were transfected with siRNAs targeting TS, TK1 and TK2 in various combinations, treated with gemcitabine (3–7.5 nM), and the effect on proliferation was measured at 96 h post-transfection as described in *Materials and Methods*. Bars indicate means ± SEM (*n* = 9–18) as a percent of proliferation of cells treated with control, non-targeting C2 siRNA without gemcitabine. *different from cells transfected with C2 siRNA but otherwise treated identically (*p* < 0.05, ANOVA).

TK2 is one of three enzymes that mediate dTMP synthesis: thymidylate synthase (TS) is responsible for *de novo* dTMP production, and TK1 is an additional thymidine salvage pathway enzyme [16, 17]. We assessed whether sensitization to gemcitabine by decreasing TK2 was due to decreasing the level of dCMP or that of dTMP. Therefore, TK2^MEDIUM^(HeLa) cells were assessed for sensitivity to gemcitabine in the context of siRNA knockdown of TS and TK1 in addition to knockdown of TK2. TK2 knockdown, and not TS or TK1 knockdown, sensitized HeLa cells to gemcitabine (Figure 4A–4D). Of 4 tested gemcitabine concentrations, TK1 reduction sensitized cells to gemcitabine at only one (6 nM), but only minimally and to a lesser degree than did reduction of TK2 (Figure 4B).

### Combined treatment with TK2 siRNA and gemcitabine decreased mitochondrial DNA content

**Figure 5:**
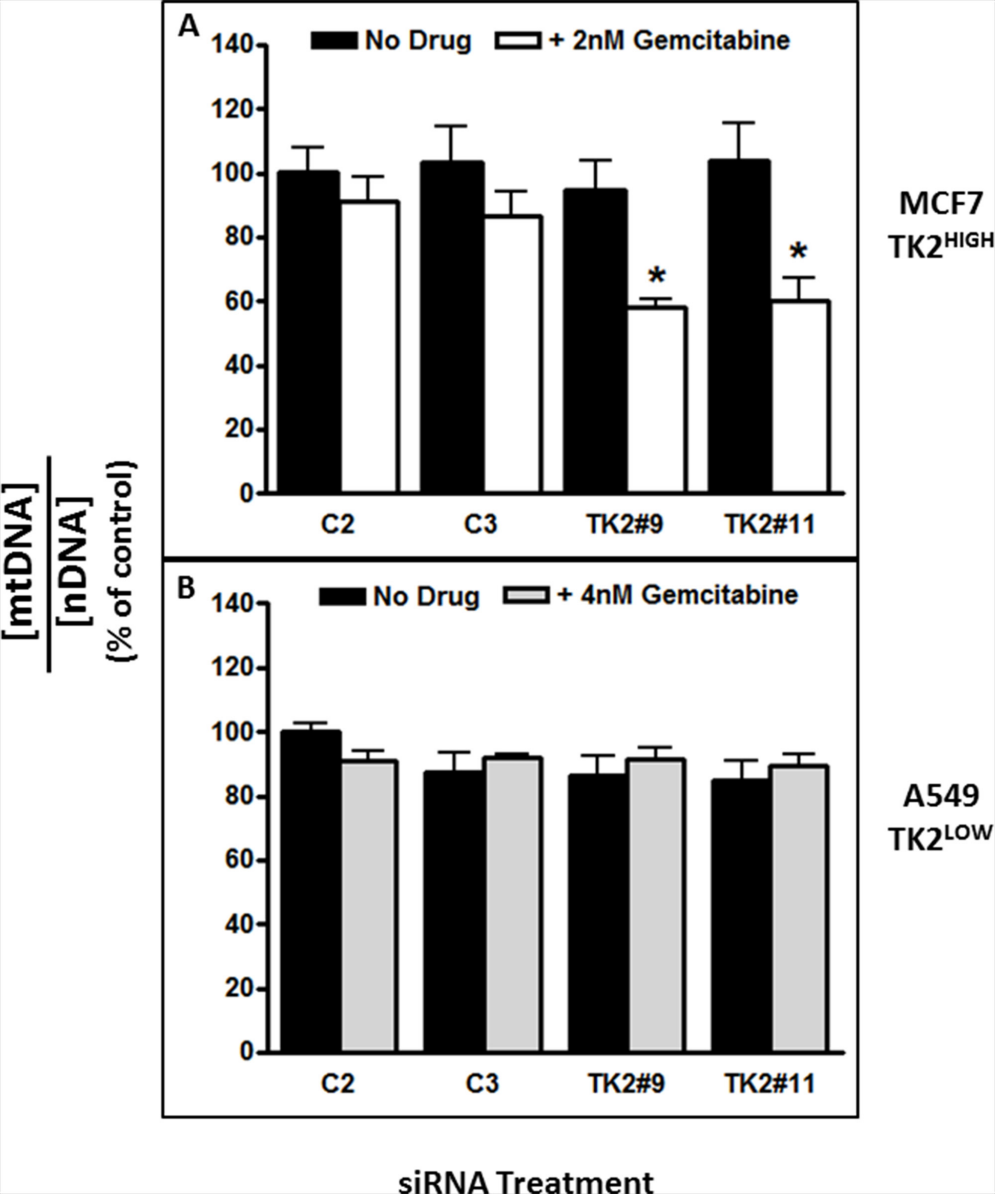
The combination of TK2 siRNA and gemcitabine decreased mitochondrial DNA content in TK2^HIGH^(MCF7) cells but not in TK2^LOW^(A549) cells MCF7 and A549 cells were transfected with siRNA, treated with gemcitabine (the IC_50_ as determined in each cell line after TK2 siRNA transfection), and the mtDNA:nDNA (mitochondrial DNA:nuclear DNA) ratio was determined 96 h later as described in *Materials and Methods*. Data are expressed as a percent of cells treated with C2 siRNA without gemcitabine. Bars indicate means ± SEM (*n* = 9). *different from cells transfected with C2 or C3 control siRNAs and otherwise treated identically (*p* < 0.05, Student's *t* test and ANOVA).

The alamarBlue assay used in the assays presented in Figure 4 is primarily dependent upon mitochondrial respiration functions including electron transport and oxidation [18]. Because TK2 is a mitochondrial enzyme, sensitization to impairment of mitochondrial function as a consequence of TK2 knockdown in the context of gemcitabine treatment was assessed. After a 96-h treatment with TK2 siRNA and gemcitabine (treated at the IC_50_, as determined in cells treated with TK2 siRNAs), total DNA was collected from TK2^HIGH^ (MCF7) and TK2^LOW^ (A549) cells, and mtDNA:nDNA ratios were assessed. TK2 siRNA-induced sensitization to gemcitabine in TK2^HIGH^ MCF7 cells (Figure 2A, 2B) was accompanied by reduction in the mtDNA:nDNA ratio (Figure 5A). There was no reduction in that ratio in identically-treated TK2^LOW^ A549 cells (Figure 5B), consistent with the lack of gemcitabine sensitization induced by TK2 siRNA in those cells (Figure 2E, 2F).

### Combined treatment with TK2 siRNA and gemcitabine decreased mitochondrial activity

Relative mtDNA content (the mtDNA:nDNA ratio) is an indirect indicator of mtDNA function and mitochondrial biogenesis and activity [19]. Mitotracker CMX ROS staining depends on intact, functional mitochondrial membrane, and the degree of staining is correlated with intact mitochondrial membrane potential and mitochondrial activity. Mitochondrial function and activity were assessed more directly using Mitotracker staining and flow cytometry.

**Figure 6:**
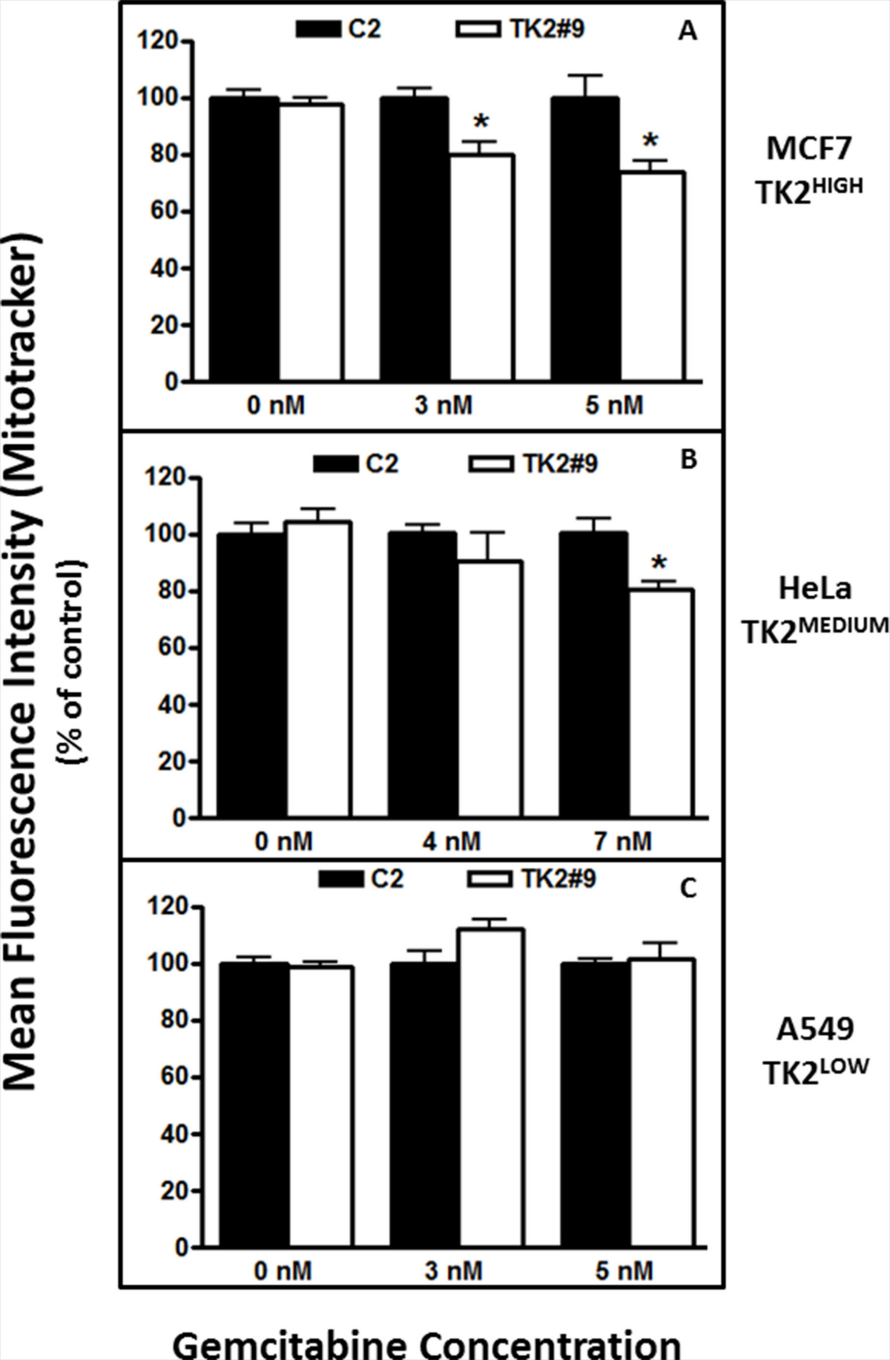
TK2 siRNA and gemcitabine decrease mitochondrial activity in TK2-expressing MCF7 and HeLa cells, but not in TK2^LOW^ A549 cells Cells were transfected with siRNAs and treated with gemcitabine for 96 h, and mitochondrial activity was measured as described in *Materials and Methods*. Bars indicate means ± SEM (*n* = 9) as a percent of the mean in cells transfected with C2 siRNA and exposed to the same gemcitabine concentration. *different from cells transfected with C2 siRNA and treated with the same concentration of gemcitabine (*p* < 0.05, Student's *t* test).

Treatment with TK2 siRNA as a single agent did not change MitoTracker staining (Figure 6). TK2 siRNA treatment decreased mitochondrial activity at both concentrations of gemcitabine in TK2^HIGH^ (MCF7) cells (Figure 6A), but only at the higher concentration of gemcitabine in TK2^MEDIUM^ (HeLa) cells (Figure 6B). TK2 siRNA treatment of the TK2^LOW^(A549) cell line did not affect mitochondrial activity in combination with gemcitabine (Figure 6C). In TK2-expressing cell lines, combined treatment with TK2 siRNA and gemcitabine decreased mitochondrial membrane potential and activity.

## DISCUSSION

Differences in achievable TK2 siRNA-induced sensitization to gemcitabine in cell lines appears, at least in part, to be due to differences in basal TK2 levels (Figure 1A). Although antisense treatment decreased TK2 protein by only about 25% in MCF7 and HeLa cells (Figure 1B, 1C), it sensitized those cells to gemcitabine by as much as 50% (Figure 2A–2D). This suggests a significant contribution of TK2 to gemcitabine resistance.

An increase in dCK levels in human tumor cells was demonstrated for the first time in response to combined treatment with TK2 siRNA and gemcitabine (but neither treatment alone) (Figure 3B, 3D). Although others have reported increased dCTP levels in response to reduced TK2 in HeLa cells [20], increased dCK levels in response to antisense targeting of TK2, particularly in combination with gemcitabine, is novel. dCK is required to activate gemcitabine and, in fact, decreased dCK levels *in vitro* mediate gemcitabine resistance [21, 22]. Higher TK2 expression may indicate greater reliance on TK2 for production of dCMP/dCTP, compared to cells with lower TK2 (for example, MCF7 and HeLa cells compared to A549 cells). When TK2-expressing cells were targeted with both TK2 siRNA and gemcitabine, dCK levels increased, possibly to compensate for decreased dCTP.

A relationship among dCK, gemcitabine, and TK2 is proposed (Figure 3E). dCK phosphorylates both deoxycytidine and the anticancer drug gemcitabine. dCK is negatively regulated by the feedback inhibition of dCTP, as is TK2. TK2 monophosphorylates both deoxythymidine and deoxycytidine, but gemcitabine is very poorly phosphorylated by TK2. Thus, TK2 contributes to levels of dCTP that negatively regulate dCK. TK2-produced dCTP would inhibit dCK and, consequently, decrease the amount of dCK available to activate gemcitabine. siRNA-mediated reduction in TK2, in combination with gemcitabine, results in increased dCK enzyme levels and likely contributes to a greater response to the antiproliferative effects of gemcitabine. Thus, TK2 contributes to gemcitabine resistance.

TK2 is one of three enzymes that mediate dTMP synthesis: in addition to the activity of TK2, thymidylate synthase (TS) is responsible for *de novo* dTMP production, and TK1 is an additional dTMP salvage pathway enzyme [16, 17]. siRNA treatments were used to decrease TS, TK1, and TK2 either singly or in combinations (both TS and TK2; both TK1 and TK2; all 3). Sensitization to gemcitabine appears to be specific to knockdown of TK2 alone and cannot be generalized to knockdown of the other dTMP-producing enzymes (TS and TK1), alone or in combination with each other.

Implications of decreased TK2 in the context of combined treatment with gemcitabine were also explored with a focus on mitochondrial toxicity. Gemcitabine can inhibit the activity of human mitochondrial DNA polymerase gamma [15]. This, in combination with a reduction in TK2, may contribute to the preferential decrease in mtDNA. Mitochondria are susceptible to the toxic effects of nucleoside analogues and patients exposed to prolonged nucleoside analogue chemotherapy, including gemcitabine, display similar pathology to a heterogeneous group of disorders (mtDNA depletion disorders) caused by mutations and deletions in the TK2 gene [23–27]. Both MCF7 and A549 cells were treated with a concentration of gemcitabine that resulted in a 50% growth inhibition in the presence of TK2 siRNA (Figure 5). The decrease in mtDNA:nDNA ratio was concomitant with sensitization to gemcitabine induced by TK2 siRNA in TK2^HIGH^ (MCF7) cells (Figure 5A), but not A549 cells with low TK2 (Figure 5B), implies that TK2 is critically involved in tumor cell mitochondrial dysfunction and subsequent toxicity in response to combined treatment with TK2 siRNA and gemcitabine in these cells. We further showed that combined treatment with TK2 siRNA and gemcitabine results in decreased mitochondrial membrane potential and activity in TK2^HIGH^- and TK2^MEDIUM^-expressing cells (Figure 6). These data implicate increased mitochondrial toxicity as a critical event in gemcitabine sensitization. Whether mitochondrial toxicity is the result of increased gemcitabine activity, or damage to mitochondria increases tumor cell sensitivity to gemcitabine, remains to be determined.

It has been suggested that TK2 plays a role in the effectiveness of deoxycytidine nucleoside analogues such as gemcitabine, and that the mitochondria could play a role in both toxicity and antitumor activity [13, 24, 28, 29]. Prior to the current study, there has been no report of the consequences of antisense-mediated TK2 reduction on gemcitabine effectiveness and mitochondrial function. This report is the first to demonstrate that TK2 mediates resistance to gemcitabine, and identifies TK2 as a potentially valuable target for anticancer therapy in combination with gemcitabine. Although siRNAs are difficult to administer effectively *in vivo* due to stability and pharmacokinetic challenges [30–32], TK2-inhibiting small molecules including those reported by others [33, 34] may be appropriate candidate TK2-targeting agents for use in combination with gemcitabine. This also sets a precedent for the potential use of other TK2-targeting antisense molecules such as phosphorothioated oligodeoxynucleotides, the clinical use of which is approved for some indications. Targeting of TK2 is likely to be of optimal therapeutic use in combination with gemcitabine or related drugs. siRNA-mediated knockdown of TK2 alone did it not reduce tumor cell proliferation, nor did it sensitize cells to pemetrexed, cisplatin, or 5FUdR [7].

We show for the first time that siRNA-mediated knockdown of TK2 preferentially sensitizes TK2-expressing cancer cell lines to the anti-proliferative effects of gemcitabine *in vitro*. Furthermore, combined treatment with TK2 siRNA and gemcitabine, in addition to increasing gemcitabine effectiveness as an antiproliferative agent, increased dCK enzyme levels. With respect to mitochondrial toxicity, combined TK2 siRNA and gemcitabine treatment: (1) decreased cellular metabolic activity, (2) preferentially decreased mitochondrial DNA (mtDNA, in the form of a decreased mtDNA:nDNA ratio), and (3) decreased mitochondrial activity. None of these effects (sensitization to gemcitabine or mitochondrial toxicity) were seen in A549 non-small cell lung cancer cells with low basal TK2 expression (7% of that in MCF7 and 25% of that in HeLa cells). Overall, these data indicate that endogenous TK2 in human tumor cells can reduce the therapeutic efficacy of gemcitabine, and antisense knockdown of TK2 increases gemcitabine activity. TK2 appears to have potential as a therapeutic target in cancer treatment and as a diagnostic biomarker of relative resistance to gemcitabine in human tumors.

## MATERIALS AND METHODS

### Human tumor cell lines

Breast epithelial adenocarcinoma (MCF7), human cervical carcinoma (HeLa), and lung epithelial carcinoma (A549) cell lines were obtained from the American Type Culture Collection (ATCC, Manassas, VA, USA). Their characteristics and maintenance information are as previously described [7], except that A549 were cultured using Alpha Modification of Eagle's Medium (AMEM) (Wisent Inc., St-Bruno, QC, Canada).

### siRNAs

All siRNAs were obtained from Dharmacon RNA Technologies-Thermo Scientific (Lafayette, CO, USA). siRNA sequences are shown in Table 1. Control siRNAs C2 and C3 contain four or more mismatches to all known human RNAs.

**Table 1:** Sequence of siRNAs used for targeting mRNA and for measuring transfection efficiency

siRNA	Targeted RNA	Target mRNA Sequence (not including -UU overhangs)	Position in mRNA Transcript
ON-TARGETplus Non-targeting siRNA #2 (Dharmacon Control #2; C2)	No Target	5′-UGGUUUACAUGUUGUGUGA-3′	
ON-TARGETplus Non-targeting siRNA #3 (Dharmacon Control #3; C3)	No Target	5′-UGGUUUACAUGUUUUCUGA-3′	
Human TK2 ON-TARGETplus siRNA(TK2 #9 siRNA)	TK2 mRNA	5′-AAAUCGGGAUCGAAUAUUA-3′	1101–1119 Coding region
Human TK2 ON-TARGETplus siRNA(TK2 #11 siRNA)	TK2 mRNA	5′-UCACAGCGCAAGAUACAUU-3′	759–777 Coding region
TYMS siGENOME siRNA (TS siRNA)	TS mRNA	5′-GGACUUGGGCCCAGUUUAU-3′	526–544 Coding region
Cy3-labelled TS #4 siRNA (Cy3-siRNA)	TS mRNA	5′-GGACUUGGGCCCAGUUUAU-3′	526–544 Coding region
Human TK1 ON-TARGETplus siRNA(TK1 siRNA)	TK1 mRNA	5′-CAAAGACACUCGCUACAGC-3′	578–596 Coding region

Cy3-labeled TS siRNA #4 has the Cy3 fluorophore ligated to the 5′ end on the antisense strand to avoid interference with the important seed region at the 3′ end

### Drugs

Gemcitabine (Gemzar, Eli Lilly and Co., Toronto, ON, Canada) was purchased from the London Regional Cancer Program pharmacy.

### siRNA Transfection

Cells were plated (3.5 × 10^5^ cells/per 25-cm^5^ flask) 24 h prior to siRNA transfection. Cells were transfected with 10 nM total siRNA using Lipofectamine 2000 (LF2K, Life Technologies, Burlington, ON, Canada) according to manufacturer's instructions [7]. Four h after transfection, cells were harvested at specified times and at no more than 75% confluence from triplicate flasks, pooled (to avoid variability in siRNA transfection) and replated into 6-well or 96-well plates, or 25-cm^5^ flasks, prior to drug treatment. In certain experiments, gemcitabine-containing medium or medium only was added to cells 4 h after replating. When TK2 #9 siRNA, TS siRNA, and TK1 siRNA were combined, respective nM concentrations were 4:3:3. When used singly or in combination with one other siRNA, the same nM concentrations were used and brought to a total of 10 nM with C2 siRNA.

### mRNA measurement

Cells collected for mRNA analysis were lysed in TRIzol Reagent (Life Technologies), and total cellular RNA was isolated according to the manufacturer's protocol. Purified RNA (1 μg) was used to synthesize cDNA by reverse transcription by MMLV-RT (Life Technologies) and random primers according to the protocol provided by the manufacturer. TK2 mRNA and GAPDH mRNA were assessed simultaneously by multiplex real-time qPCR amplification using a TaqMan Gene Expression Assay kit (Applied Biosystems, Life Technologies Holdings, Burlington, Canada) as described previously [7]. A VIC-labelled GAPDH expression assay (Applied Biosystems) was used to measure GAPDH mRNA. Reactions products were measured using a ViiA 7™ Real-time PCR System (Applied Biosystems).

### Protein measurement

Total cell protein lysates were obtained at the indicated times and sonicated at 4°C using a Vibra Cell™ ultrasonic processor (Sonics & Materials Inc., Danbury, CT). Protein was estimated using a Bio-Rad Protein Assay kit (Bio-Rad, Mississauga, ON, Canada) and the manufacturer's protocol.

Total protein (35 μg) was separated using a SDS-PAGE (15% gel) and transferred to a nitrocellulose membrane (Hybond-ECL, GE Healthcare Biosciences, Cedarlane, Burlington, ON, Canada) as described previously [7]. TK2, dCK, and actin proteins were visualized using rabbit anti-human TK2 antibody (HPA041162, Sigma-Aldrich at 1:12,000 in 1% skim milk in TBS-T), rabbit anti-human dCK antibody (ab83046, Abcam, 1:400 in 5% BSA in TBS-T), and rabbit anti-human actin antibody (Sigma-Aldrich, 1:1000 in 1% skim milk in TBS-T). Bands were detected using horseradish peroxidase-conjugate anti-rabbit secondary antibody (GE Healthcare Biosciences, 1:10,000) on a STORM 860 Molecular Imager (Amersham Biotech-Molecular Dynamics Inc, Sunnyvale, CA, USA). Band intensity was quantified using ImageQuant 5.1 software (Amersham Biotech-Molecular Dynamics Inc.). TK2 and dCK were normalized to the actin internal standard and then as a percent of relative amounts in cells transfected with control, non-targeting C2 siRNA.

### Cell proliferation measurements

#### Cell counting

Tumor cell proliferation after treatment with siRNAs and/or gemcitabine was measured as described previously [7]. Briefly, cells were washed with PBS, trypsinized, and counted on a Beckman Coulter Z1 Particle Counter (Beckman, Mississauga, Ontario, Canada) 96 h after treatment. The fold change in cell number was determined after 4 days of growth, and presented as a percent of that of control cultures.

#### Cell viability staining assay

After siRNA transfection, cells were replated into 96- well plates and treated, in triplicate, with drug or control medium. Relative cell density after 96 h was determined using the vital stainalamarBlue and measuring fluorescence (595 nm) using a Wallac Victor^2^1420 multilabel counter (PerkinElmer, Woodbridge, ON, Canada).

### Mitochondrial and nuclear DNA (mtDNA and nDNA) measurement

Mitochondrial and nuclear DNA target genes, DNA isolation, and primer and probe sequences for quantitative PCR assessment of relative nuclear and mitochondrial DNA content were as described previously [35–39]. Total DNA was isolated from cells, and relative mtDNA and nDNA in 300 ng of total DNA were estimated by quantitative PCR using TaqMan primer-probe sets specific to mtDNA (mitochondrial NADH dehydrogenase 1 [NADHD1]) and nDNA (18S rRNA) target genes. Reaction products were amplified using the ViiA 7™ Real-time PCR System.

### Flow cytometry

Cells were trypsinized, resuspended in media with 10% FBS, washed twice in PBS and precipitated by centrifugation. When transfection efficiency was assessed in cells transfected with Cy3-labeled siRNA, cells were harvested 4 h post-transfection and resuspended in 400 μl PBS after the final PBS wash. When mitochondrial number/activity was assessed using Mitotracker® Red CMXRos (Invitrogen), cells were incubated in Mitotracker (200 nM, 37°C, 20 min) prior to washing in PBS and resuspendion in PBS (400 μl). Cy3 fluorescence or Mitotracker signal were assessed in unfixed cells using a BD FACSCalibur flow cytometer (Becton-Dickenson, Mississauga, Canada). Relative fluorescence intensity was analyzed using FlowJo V10 software.

### Statistical analysis

Data are presented as means ± SEM. ANOVA (when measuring differences among multiple means) or Student's *t* test (when measuring differences between two means) were used to determine significance. The level of significance for all statistical analyses was chosen *a priori* to be *p* < 0.05.

## SUPPLEMENTARY FIGURES



## References

[1] Galmarini CM, Mackey JR, Dumontet C (2001). Nucleoside analogues: mechanisms of drug resistance and reversal strategies. Leukemia.

[2] Arisan ED, Obakan P, Coker-Gurkan A, Calcabrini A, Agostinelli E, Unsal NP (2014). CDK inhibitors induce mitochondria-mediated apoptosis through the activation of polyamine catabolic pathway in LNCaP, DU145 and PC3 prostate cancer cells. Curr Pharm Des.

[3] Chen EH, Johnson EE, Vetter SM, Mitchell BS (1995). Characterization of the deoxycytidine kinase promoter in human lymphoblast cell lines. J Clin Invest.

[4] Munch-Petersen B, Cloos L, Tyrsted G, Eriksson S (1991). Diverging substrate specificity of pure human thymidine kinases 1 and 2 against antiviral dideoxynucleosides. J Biol Chem.

[5] Munch-Petersen B, Knecht W, Lenz C, Sondergaard L, Piskur J (2000). Functional expression of a multisubstrate deoxyribonucleoside kinase from Drosophila melanogaster and its C-terminal deletion mutants. J Biol Chem.

[6] Al-Madhoun AS, Tjarks W, Eriksson S (2004). The role of thymidine kinases in the activation of pyrimidine nucleoside analogues. Mini Rev Med Chem.

[7] Di Cresce C, Figueredo R, Ferguson PJ, Vincent MD, Koropatnick J (2011). Combining siRNAs targeting thymidylate synthase and thymidine kinase 1 or 2 sensitizes human tumor cells to 5FUdR and pemetrexed. J Pharmacol Exp Ther.

[8] Gesto DS, Cerqueira NM, Fernandes PA, Ramos MJ (2012). Gemcitabine: a critical nucleoside for cancer therapy. Curr Med Chem.

[9] Huang P, Chubb S, Hertel LW, Grindey GB, Plunkett W (1991). Action of 2′, 2′-difluorodeoxycytidine on DNA synthesis. Cancer Res.

[10] Wang J, Lohman GJ, Stubbe J (2007). Enhanced subunit interactions with gemcitabine-5′-diphosphate inhibit ribonucleotide reductases. Proc Natl Acad Sci U S A.

[11] Heinemann V, Hertel LW, Grindey GB, Plunkett W (1988). Comparison of the cellular pharmacokinetics and toxicity of 2′, 2′-difluorodeoxycytidine and 1-beta-D-arabinofuranosylcytosine. Cancer Res.

[12] Jansson O, Eriksson S (1990). Direct photoaffinity-labelling of human deoxycytidine kinase with the feedback inhibitor dCTP. Biochem J.

[13] Damaraju S, Damaraju VL, Mowles D, Sawyer MB, Cass CE (2010). Cytotoxic activity of gemcitabine in cultured cell lines derived from histologically different types of bladder cancer: role of thymidine kinase 2. Biochem Pharmacol.

[14] Wang L, Munch-Petersen B, Herrstrom Sjoberg A, Hellman U, Bergman T, Jornvall H, Eriksson S (1999). Human thymidine kinase 2: molecular cloning and characterisation of the enzyme activity with antiviral and cytostatic nucleoside substrates. FEBS Lett.

[15] Fowler JD, Brown JA, Johnson KA, Suo Z (2008). Kinetic investigation of the inhibitory effect of gemcitabine on DNA polymerization catalyzed by human mitochondrial DNA polymerase. J Biol Chem.

[16] Hu CM, Chang ZF (2007). Mitotic control of dTTP pool: a necessity or coincidence?. J Biomed Sci.

[17] Carreras CW, Santi DV (1995). The catalytic mechanism and structure of thymidylate synthase. Annu Rev Biochem.

[18] Springer JE, Azbill RD, Carlson SL (1998). A rapid and sensitive assay for measuring mitochondrial metabolic activity in isolated neural tissue. Brain Res Brain Res Protoc.

[19] Ralph SJ, Rodriguez-Enriquez S, Neuzil J, Saavedra E, Moreno-Sanchez R (2010). The causes of cancer revisited: “mitochondrial malignancy” and ROS-induced oncogenic transformation - why mitochondria are targets for cancer ther apy. Mol Aspects Med.

[20] Desler C, Munch-Petersen B, Rasmussen LJ (2006). The role of mitochondrial dNTP levels in cells with reduced TK2 activity. Nucleosides Nucleotides Nucleic Acids.

[21] Nakano Y, Tanno S, Koizumi K, Nishikawa T, Nakamura K, Minoguchi M, Izawa T, Mizukami Y, Okumura T, Kohgo Y (2007). Gemcitabine chemoresistance and molecular markers associated with gemcitabine transport and metabolism in human pancreatic cancer cells. Br J Cancer.

[22] Galmarini CM, Clarke ML, Jordheim L, Santos CL, Cros E, Mackey JR, Dumontet C (2004). Resistance to gemcitabine in a human follicular lymphoma cell line is due to partial deletion of the deoxycytidine kinase gene. BMC Pharmacol.

[23] Saada A (2004). Deoxyribonucleotides and disorders of mitochondrial DNA integrity. DNA Cell Biol.

[24] Priego EM, Karlsson A, Gago F, Camarasa MJ, Balzarini J, Perez-Perez MJ (2012). Recent Advances in Thymidine Kinase 2 (TK2) Inhibitors and New Perspectives for Potential Applications. Curr Pharm Des.

[25] Lewis W, Dalakas MC (1995). Mitochondrial toxicity of antiviral drugs. Nat Med.

[26] Saada A, Shaag A, Mandel H, Nevo Y, Eriksson S, Elpeleg O (2001). Mutant mitochondrial thymidine kinase in mitochondrial DNA depletion myopathy. Nat Genet.

[27] Wang L, Saada A, Eriksson S (2003). Kinetic properties of mutant human thymidine kinase 2 suggest a mechanism for mitochondrial DNA depletion myopathy. J Biol Chem.

[28] Nielsen SE, Munch-Petersen B, Mejer J (1995). Increased ratio between deoxycytidine kinase and thymidine kinase 2 in CLL lymphocytes compared to normal lymphocytes. Leuk Res.

[29] Bergman AM, Pinedo HM, Peters GJ (2002). Determinants of resistance to 2′, 2′-difluorodeoxycytidine (gemcitabine). Drug Resist Updat.

[30] Dykxhoorn DM, Lieberman J (2006). Running interference: prospects and obstacles to using small interfering RNAs as small molecule drugs. Annu Rev Biomed Eng.

[31] Dominska M, Dykxhoorn DM (2010). Breaking down the barriers: siRNA delivery and endosome escape. J Cell Sci.

[32] Rivera S, Yuan F (2012). Critical issues in delivery of RNAi therapeutics *in vivo*. Curr Pharm Biotechnol.

[33] Balzarini J, Hernandez AI, Roche P, Esnouf R, Karlsson A, Camarasa MJ, Perez-Perez MJ (2003). Non-nucleoside inhibitors of mitochondrial thymidine kinase (TK-2) differentially inhibit the closely related herpes simplex virus type 1 TK and Drosophila melanogaster multifunctional deoxynucleoside kinase. Mol Pharmacol.

[34] Perez-Perez MJ, Hernandez AI, Priego EM, Rodriguez-Barrios F, Gago F, Camarasa MJ, Balzarini J (2005). Mitochondrial thymidine kinase inhibitors. Curr Top Med Chem.

[35] Gianotti TF, Sookoian S, Dieuzeide G, Garcia SI, Gemma C, Gonzalez CD, Pirola CJ (2008). A decreased mitochondrial DNA content is related to insulin resistance in adolescents. Obesity (Silver Spring).

[36] Bai RK, Perng CL, Hsu CH, Wong LJ (2004). Quantitative PCR analysis of mitochondrial DNA content in patients with mitochondrial disease. Ann N Y Acad Sci.

[37] (2001). Extraction and precipitation of DNA. Curr Protoc Hum Genet.

[38] Wong L-JC, Lam C-W (1997). Alternative, Noninvasive Tissues for Quantitative Screening of Mutant Mitochondrial DNA. Clinical Chemistry.

[39] Venegas V, Wang J, Dimmock D, Wong LJ (2011). Real-time quantitative PCR analysis of mitochondrial DNA content. Curr Protoc Hum Genet.

