# Supplementary material for: siRNA knockdown of mitochondrial thymidine kinase 2 (TK2) sensitizes human tumor cells to gemcitabine
Source: Oncotarget. 2015 Jun 15;6(26):22397–409. doi: 10.18632/oncotarget.4272 (PMC4673171; doi:10.18632/oncotarget.4272)
Supplement: Supplementary file 1 [file oncotarget-06-22397-s001.pdf]

SUPPLEMENTARY FIGURES

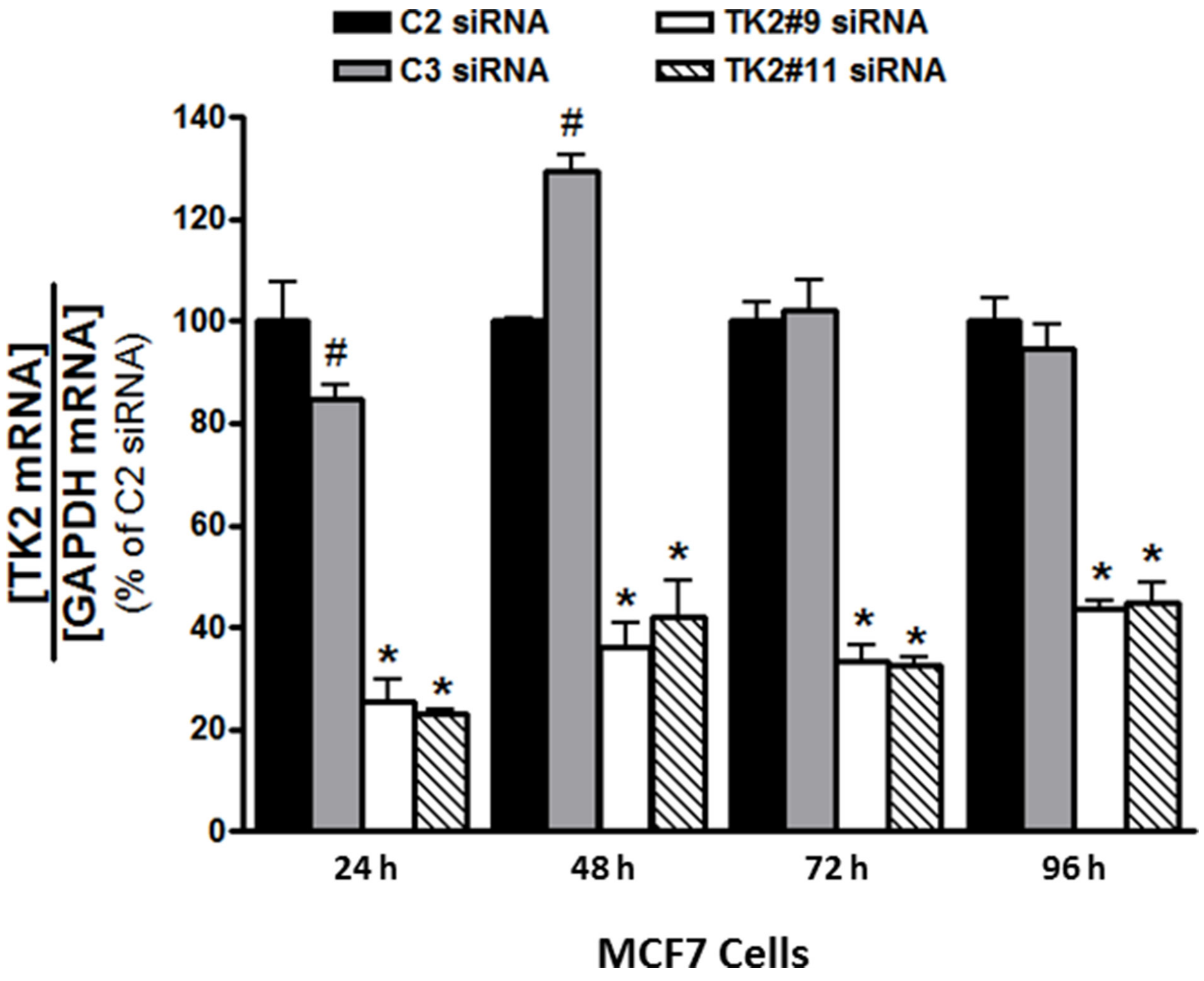

Supplementary Figure S1: siRNA-mediated knockdown of TK2 mRNA in MCF7 cells, 24–96 h post-transfection with 10 nM siRNA. Relative TK2 mRNA is shown as a percent of the amount in cells transfected with control, non-targeting C2 siRNA. Bars represent means ± SEM ($n = 9$). *different from cells treated with C2 or C3 siRNA ($p < 0.05$ Student's t test and/or ANOVA). [#]different from cells treated with C2 siRNA ($p < 0.05$ Student's t test and/or ANOVA).

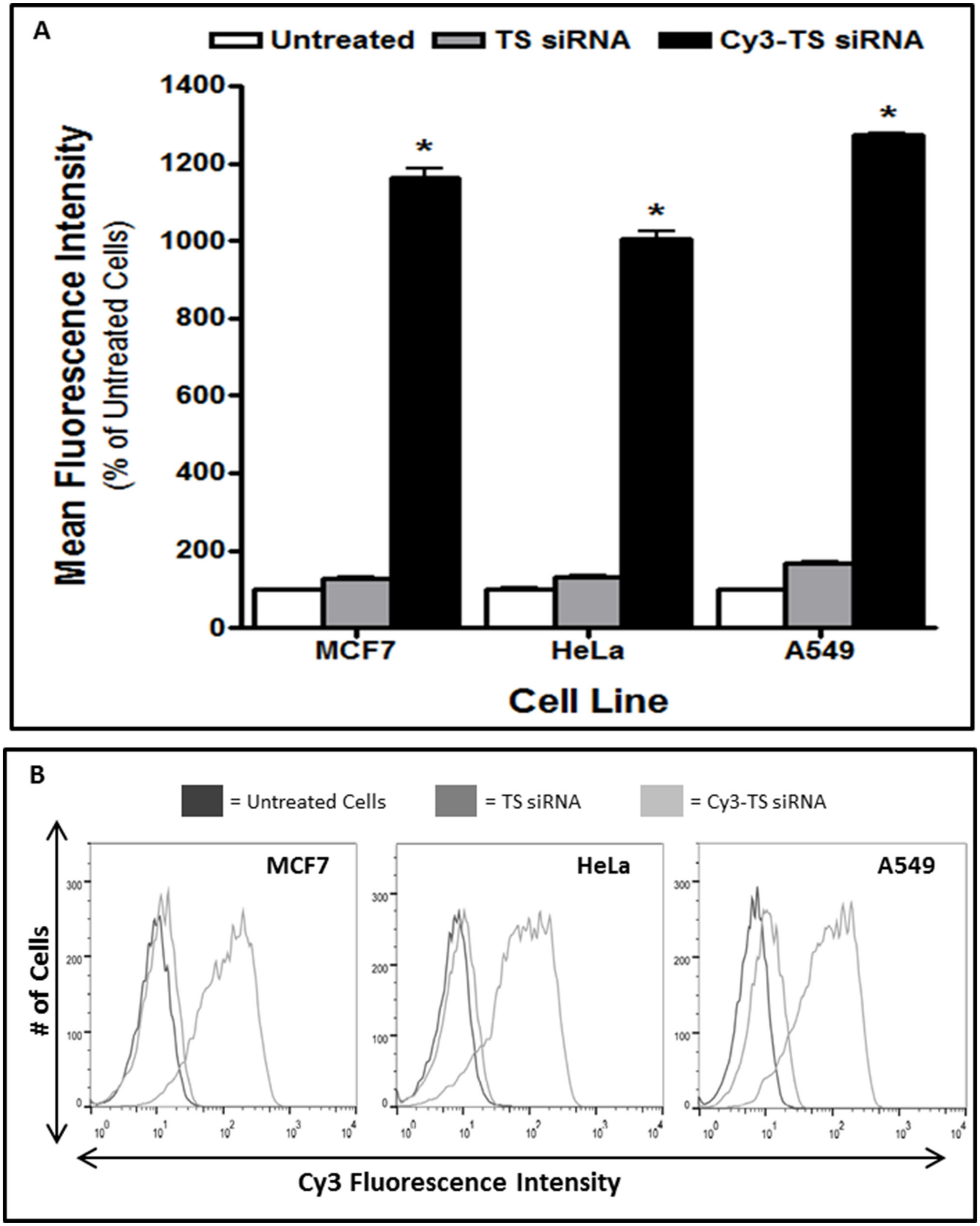

Supplementary Figure S2: siRNA transfection efficiency in TK2^{HIGH}(MCF7), TK2^{MEDIUM}(HeLa), and TK2^{LOW}(A549) cells. Cells were untransfected (white bars), transfected with unlabeled TS siRNA (grey bars), or transfected with Cy3-labeled TS siRNA (black bars). Mean fluorescence intensity (MFI) was determined by flow cytometry 4 h post-transfection with 10 nM siRNA using LF2K. Data are shown as the percent increase in MFI compared to untransfected cells. Bars represent means ± SEM (n = 9). *different from untransfected cells or cells transfected with unlabeled TS siRNA cells ($p < 0.05$, Student's t test or ANOVA).

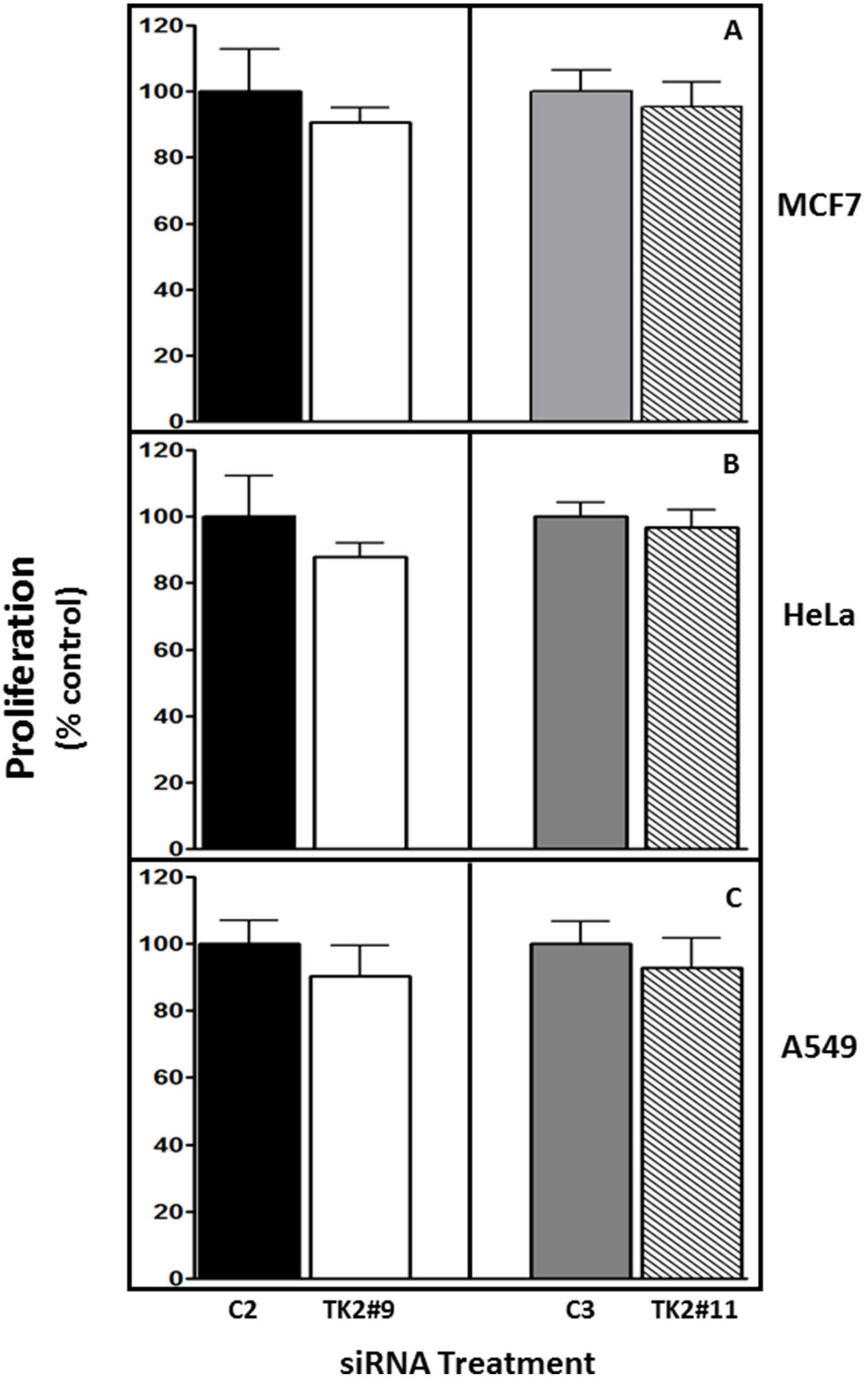

Supplementary Figure S3: TK2 siRNAs alone do not affect proliferation of tumor cell lines in this study. Cells were transfected with control non-targeting or TK2-targeting siRNAs. Proliferation was measured by cell counting as described in *Materials and Methods*, and presented as a percent of that of cells treated with control, non-targeting siRNA (C2 for TK2#9 siRNA, C3 for TK2#11 siRNA). Bars represent means ± SEM ($n = 9$).

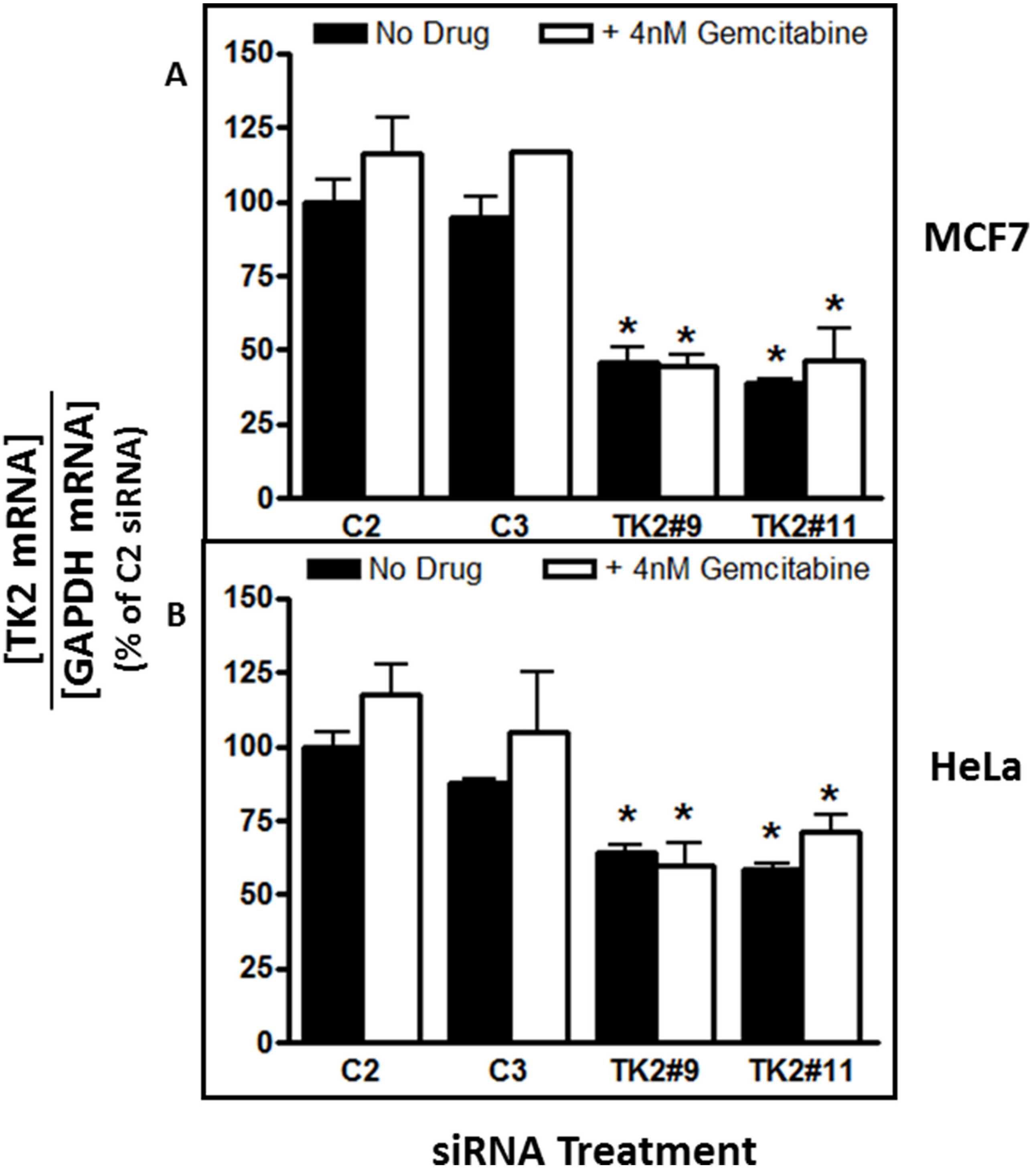

Supplementary Figure S4: TK2 siRNA reduces TK2 mRNA (same cells for which data are shown in Figure 3). MCF7 **A.** and HeLa **B.** cells were transfected with siRNA, treated with gemcitabine, and relative TK2 mRNA levels were measured 96 h post-transfection as described in *Materials and Methods*. TK2 and dCK protein were measured in the same experiment, and those data are presented in Figure 3. Data are expressed as a percent of non-targeting control C2 siRNA without drug. Bars represent mean ± SEM ($n = 9$). *different from cells transfected with C2 or C3 siRNA ($p < 0.05$, Student's t test or ANOVA).
